# Prophylactic Antibiotics, the Mediator of Post-Stroke Infections: A Systematic Review

**DOI:** 10.7759/cureus.15055

**Published:** 2021-05-16

**Authors:** Andrew Ndakotsu, Revathi Myneni, Aimen Iqbal, Amit S Grewal, Ansha P Abubacker, Govinathan Vivekanandan, Harsh V Chawla, Safeera Khan

**Affiliations:** 1 Internal Medicine, California Institute of Behavioral Neurosciences & Psychology, Fairfield, USA; 2 Research, California Institute of Behavioral Neurosciences & Psychology, Fairfield, USA; 3 Emergency Medicine, California Institute of Behavioral Neurosciences & Psychology, Fairfield, USA

**Keywords:** prophylactic antibiotics, post-stroke, infections, anti-bacterial agents, stroke, stroke associated pneumonia

## Abstract

Infections frequently complicate an acute stroke and have been associated with an unfavorable prognosis among patients. The use of prophylactic antibiotics seems rational, however, its efficacy has remained obscure. This systematic review aims to offer more clarity to this established dilemma. PubMed and Google Scholar were explored to gain access to studies on post-stroke infection. A systematic review was carried out to analyze how profitable it would be to offer preventive antibiotics immediately after an acute stroke. Five randomized control trials were obtained and analyzed the efficacy of antibiotics in acute stroke according to their intrinsic effects on the infection rate, functionality, and mortality benefits. Based on our findings, we discovered that antibiotics reduce the onset of early infections, especially urinary tract infections, but have absolutely no effect on the functionality and offer no mortality benefit. These results were emphatically shown in two large, open-labeled randomized controlled trials involved in this systematic review. Prophylactic antibiotics provide no additional benefits to the standard of care and should not be used following an acute stroke. They may decrease the incidence of acute infections, especially urinary tract infections, but have no effects on functional outcome and mortality.

## Introduction and background

A stroke occurs when there is an impedance to the blood supply to parts of the brain, depriving the brain tissues of oxygen and vital elements, leading to subsequent cell death of the impacted region. Stroke is a prime sponsor of disability and death globally [1]. The prognosis of stroke patients is influenced by the primary brain injury and accompanying medical complications [2]. Post-stroke infection is one of these medical complications and frequently complicates the recovery following a stroke. It has also been strongly linked with poor clinical outcomes and mortality [3]. Post-stroke infections account for about 30% of complications encountered following a stroke, out of which pneumonia and urinary tract infections were the most common. Pneumonia was found to complicate about 10% of all acute stroke, out of which 50% occurred within the first 48 hours [4]. However, the incidence of pneumonia was increased in patients who possess a greater severity of stroke and incapacity [4]. An observational study that included a total of 9238 patients conducted over twelve years demonstrated a correlation between stroke-associated pneumonia and mortality, with an increase in mortality seen within one year. Hence, active management to address the risk factors that make the stroke population liable to infections cannot be over-emphasized [5,6].

Factors that have contributed immensely to the incidence of infections post-stroke includes micro-aspiration due to impaired swallowing, prolonged supine positioning, stroke-induced immunodepression syndrome [7], urinary catheterization [8], increased bladder post-void residual (PVR) [9], and translocation and dissemination of commensal bacteria [10]. Attempts to combat these risk factors have been made in recent times, one of which was using a beta-blocker to modulate the sympathetic nervous system's role in stroke-induced immunodepression syndrome. A cohort study involving 306 stroke patients was done in 2018; it was demonstrated that beta-blockers did not lower the incidence of post-stroke infections but were linked with urinary tract infections in those with insula/anteromedial strokes [11]. Recently, it has been demonstrated that Acetylcholine production is increased inside lymphocytes post an acute stroke. Interestingly, the levels of acetylcholine in the first 24 hours correlated with one-year mortality. This suggests a probable cholinergic mediated effect on the immune system [12]. This finding warrants more investigation as we seek to reduce the incidence of post-stroke infections.

Another modality that has been suggested is the prospective use of prophylactic antibacterial agents in stroke patients. In doing so, we should reduce mortality and optimize our stroke survivor's functionality, contributing immensely to the working population at large. Several studies like the preventive antibiotic in stroke study (PASS) and Stroke-INF trial have tried to appreciate the benefits of prophylactic antibiotics in arresting these infections to reduce the disabilities suffered by stroke survivors and also reducing mortality. Still, they have, however, yielded contrasting results [13,14]. Our systematic review intends to support this vulnerable population by harmonizing and analyzing these studies to develop answers and recommendations on reducing the onset and unwanted sequelae of post-stroke infections.

## Review

Post-stroke infections remain a leading cause of morbidity and mortality. The prophylactic use of antibiotics seems rational to prevent these infections. This article aims to provide evidence of whether the use of prophylactic antibiotics immediately after a stroke is justified. The selected articles were systematically reviewed, and their respective methods and results are interpreted.

Method

This systematic review was designed, and its results reported using the Preferred Reporting Items for Systematic Reviews and Meta-Analysis (PRISMA) guidelines and principles were adhered to [15].

Search strategy

Two databases, PubMed and Google Scholar were thoroughly explored electronically, employing the use of appropriate keywords and medical subject headings (MeSH) terms to precisely mine out all potentially relevant articles demonstrating the role of prophylactic antibiotics in mitigating post-stroke infections. The keywords used include stroke, cerebrovascular event, brain ischemia, antibiotics, antibacterial agents, prophylactic antibiotics, cephalosporin, infections, sepsis, and pneumonia. The Boolean scheme was used to galvanize the keywords and MeSH strategy format and subsequently employed in PubMed. All articles were subsequently retrieved, and references were thoroughly checked to avoid neglecting potentially relevant articles, there-after the titles, abstracts, and subject headings were scrutinized for relevance.

Inclusion and exclusion criteria

We restricted our choice of studies to randomized control trials, systematic reviews, and meta-analyses published during 2005-2020. Among the studies chosen, we ensured that all patients included were aseptic before the onset of the study and at least 18 years of age. In addition, only studies published in the English language were included. The population, intervention, comparison, outcomes, and study criteria (PICOS) were the fulcrum behind our eligibility criteria.

Data extraction

Data selection and extraction were carried out autonomously by two researchers (AN, RM) independently. In cases of disagreements, both researchers discussed the study design, relevance to our inclusion and exclusion criteria, intervention used, and outcome measured in other to reach a concord. In instances when common ground could not be attained, we solicited the aid of a third reviewer.

Analysis of study quality

The clinical trials were critically evaluated with the Cochrane risk of bias tool, while the systematic reviews were subjected to assessment of multiple systematic reviews (AMSTAR) [16]. With the Cochrane risk of bias tool, each study was scrutinized based on seven criteria to fish out potential biases. Each criterion was scored as either high quality, low quality, or unclear. A summary of the Cochrane risk of bias tool is given in Table 1 below.

**Table 1:** A tabulated summary of the Cochrane risk of bias tool

Trait of paper	Chamorro et al., 2005 [17]	Harms et al., 2008 [18]	Schwarz et al., 2008 [19]	Westendorp et al., 2015 [13]	Kalra et al., 2015 [14]
Random sequence generation. (Selection bias)	Low risk	Low risk	Low risk	Low risk	Low risk
Allocation of concealment. (Selection bias)	Low risk	Low risk	High risk	High risk	Low risk
Blinding of both the participants and evaluators. (Performance bias)	Low risk	Low risk	High risk	High risk	High risk
Blinding of assessment during outcome collection. (Detection bias)	Low risk	Low risk	Low risk	Low risk	Low risk
Incomplete outcome data. (Attrition bias)	High risk	Low risk	Low risk	Low risk	Low risk
Selective reporting. (Reporting bias)	Low risk	Low risk	Low risk	Low risk	Low risk
Other bias	Low risk	Low risk	Low risk	Low risk	Low risk

Similarly, the systematic reviews were subjected to quality appraisal via AMSTAR. Through this medium, we were able to access the intrinsic methodological quality of the research papers, with a score of eight and above being the benchmark for inclusion.

Results

**Figure 1:**
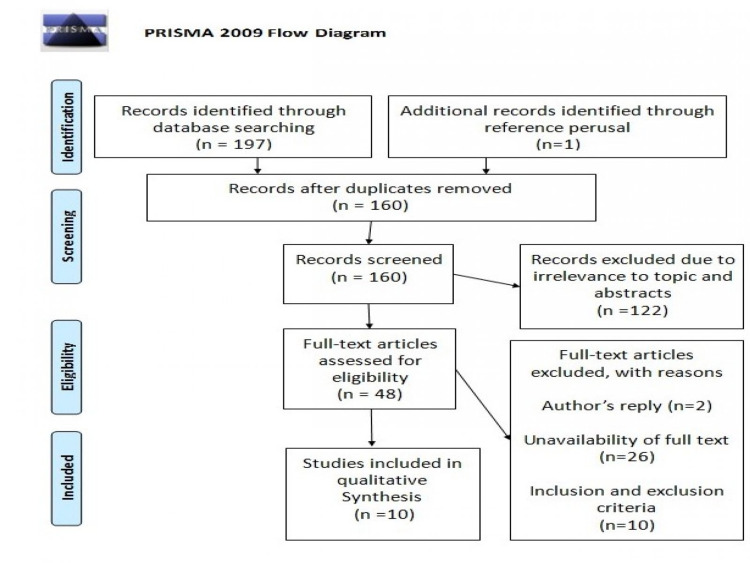
Preferred Reporting Items for Systematic Reviews and Meta-Analyses (PRISMA) flow diagram Source: Ref. [15]

A total of 198 articles were identified using the various search strategies employed. Out of 198 articles, 140 articles were from PubMed, 57 studies from Google Scholar, and one article was obtained via reference perusal; 160 articles remained after the removal of 38 duplicate articles. We then filtered the remaining articles based on the relevance of the title and contents of their respective abstracts to our ongoing research. Out of which, 112 articles were discarded due to irrelevance. Hence, 48 articles were left and we checked for availability of full texts, out of which 26 articles were removed. Two articles were found to be an author's reply and were subsequently removed. Out of the remaining articles, 10 were found eligible based on the eligibility criteria. Five clinical trials and five systematic reviews were finalized. A complete Preferred Reporting Items for Systematic Reviews and Meta-Analyses (PRISMA) flow diagram is shown below in Figure 1.

Each of the clinical trials varied in study designs, interventions, and outcomes measured. However, the goal of the studies was similar. We can see a tabulated summary of the clinical trials below in Table 2.

**Table 2:** A tabulated summary of the clinical trials n = number of patients; RCT = randomized control trials; NIHSS = National Institute of Health Stroke Scale

Authors	Study design	Inclusion criteria	Intervention	Primary outcomes	Secondary outcomes	Conclusions
Chamorro et al. [17]	A randomized, double-blinded, placebo-controlled trial	Ischemic or hemorrhagic stroke < twenty-four hours, n =136	Intravenous levofloxacin, 500 mg/100 ml/dL for three days, and placebo, [0.9% sodium chloride solution]	Rate of infections	Neurological outcomes and mortality benefits	After a period of 90 days, there was no significant difference between both groups.
Schwarz et al. [19]	Open-label randomized control trial	Ischemic stroke occurring within 24 hours. n=60	Intravenous mezlocillin(2g), plus (1g) sulbactam, eight hourly for four days.	Onset and incidence of fever.	Rate of infection and clinical outcome	They decreased body temperature signifying a reduction in the rate of infection and also a better clinical outcome.
Westendorp et al. [13]	A randomized, open-label trial	Ischemic/hemorrhagic, n = 2,538	Intravenous ceftriaxone 24 hourly for four days.	Functional outcome at three months	Death, Infection rates, length of hospital stay.	Functional outcome was not improved at the end of three months.
Karla et al. [14]	Open-label, cluster-RCT	Ischemic or hemorrhagic stroke, dysphagia, within forty-eight hours post-stroke. n=1217	Antibiotic therapy (different substances) over seven days	Pneumonia onset was less than fourteen days.	Neurological outcome	After 90 days, the was a difference in the onset of pneumonia and functional outcomes.
Harms et al. [18]	Double-blinded randomized control trial	Non-lacunar ischemic stroke, Middle cerebral Artery territory, NIHSS greater or equal to 11. n=79	Intravenous moxifloxacin, 400 mg for five days.	The onset of infection less than eleven days	No secondary outcome was measured.	There was no improvement in functional outcomes after 180 days but a lower infection rate in the treatment group.

Discussion

Current medical guidelines have continuously omitted the use of prophylactic antibiotics in the management of acute stroke despite its theoretical benefits in the prevention of post-stroke infections. Prospective studies have demonstrated a worse clinical prognosis in patients who suffer an infection secondary to an acute stroke. In order to examine this hypothesis, we will be cross-examining the above studies. We will analyze them based on their ability to reduce the incidence of infections following an acute stroke, effects on functional outcome, and mortality benefits.

Incidence of infections

It is imperative to highlight that all of the studies employed different criteria in their diagnosis of infections, and this will be summarized in Table 3 below.

**Table 3:** Criteria for infection in each of the respective clinical trials

Author	Criteria
Westendorp et al. [13]	Infection of the unclear origin or other infections. Clinical evidence of an infection whose origin is unknown or any systemic infection. The modified Centers for Disease Control and Prevention criteria (2008) was used as an Epicenter.
Karla et al. [14	A patient's temperature of greater or equal to 37.5℃ on two successive measurements or a single measurement of 38.0℃ or higher and (b) a respiratory rate of 20 cycles per min or more, or cough and breathlessness, or purulent sputum, and (c) a leucocyte count that is higher than 11.0×10⁹/L or infiltrates on chest X-ray, or positive culture of the sputum or microbiology or blood culture is demonstrating organisms.
Schwarz et al. [19]	Pneumonia: Features in keeping with a new infiltrate on the chest radiograph compatible with a diagnosis of infection plus at least one of the following findings: fever (temperature of at least 38.0℃), elevated leucocytes greater than 12,000/L or leukopenia less than 3000/L, purulent tracheal secretions. Inflammation of the trachea and bronchial tree tracheal secretions that are purulent in character or sputum plus at least one of the following findings: fever (of at least 38.0℃), elevated leucocytes greater than 12,000/L or decreased leucocytes, less than 3000/L. Urinary tract infection Evidence of greater than 25 leukocytes/L in the urine if not explained by other findings (i.e., blood sample contamination); each urinary tract infection in this patient group is considered significant bacteria in blood. Positive bacteria cultures. Sepsis clinical signs of an infection with at least two of the following: temperature 38℃ or 35℃, tachycardia is greater than 90 beats per minute. Tachypnea is greater than 20 cycles per minute. Elevated leucocytes of above 12,000/L or decreased leucocytes of lower than 3000/L.
Harms et al. [18]	Abnormal respiratory examination, pulmonary infiltrates on chest radiographs. Cough that is productive of purulent sputum, microbiological cultures from the lower respiratory tract or blood cultures, leukocytosis, and a rise in C reactive protein. Diagnosis of urinary tract infection was based on two of the following criteria: fever (＞38℃), urine sample positive for nitrite, leukocytes in urine, and significant bacteriuria.
Chamorro et al. [17]	The temperature of 37.5℃ in two measurements or 37.8℃ in a single measurement in patients with suggestive symptoms (i.e., cough, dyspnea, pleuritic pain, urinary tract symptoms), white blood cell counts 11,000/mm^3^ or 4000/mm^3^, Pulmonary infiltrate on chest X-rays or cultures demonstrating the agent of infection.

Between 2005 and 2007, we saw two clinical trials investigating the role of prophylactic antibiotics among the post-stroke populace. Both studies had similar designs as both were double-blinded and randomized. They also used antibiotics belonging to the same family (fluoroquinolones), and the primary outcome from both studies was the incidence of infections. Both antibiotics were administered intravenously, and the rate of infection was judged for less than 7 days in the study by Chamarro et al. and less than 11 days in the one conducted by Harms et al. [17,18]. They, however, differed in inclusion criteria, as shown in Table 2. Contrasting results were revealed as Chamarro et al. could not appreciate any difference between the treatment group and the standard of care group, except for increased respiratory infections among patients with a chronic obstructive disease background. Furthermore, no difference was observed among smokers [17], a feature not seen by Harms et al. [18]. It is worth noting that both groups were stratified and matched equally, eliminating any potential confounders [17]. The treatment group was also associated with a poor clinical outcome after 90 days which could be attributed to the inclusion of ischemic and hemorrhagic stroke in the study. The suggested mechanism by the authors was that levofloxacin might have some neurotoxic effects on the brain tissue. The use of moxifloxacin by Harms et al. yielded a decrease in the treatment group's infection rate in contrast to those in the standard of care group. They attributed this to its wide spectrum of antibiotic activity being a fourth-generation fluoroquinolone [18]. Clinical outcome after 180 days reflected no appreciable difference in both groups. One may, however, call to attention that the study population of both clinical trials was not enough (250 patients) to give credence to their respective inclusion of prophylactic antibiotics. Although contrasting conclusions, this difference in conclusions may be attributed to the difference that existed between the threshold of inclusion of acute stroke patients. The threshold of inclusion of patients in both studies was based on patient severity according to the National Institute of Health Stroke Scale (NIHSS) criteria (NIHSS > 5 vs. >11).

Three open-labeled randomized control trials were also conducted, although their open nature limited this study design [13,14,19]. The smallest of the trials published by Schwarz et al. used only 60 patients severely affected by acute stroke (Modified Rankin Scale greater than three). The antibiotic of choice was mezlocillin + sulbactam. However, the primary outcome was modified, restricting the infection rate measurement to the incidence and height of fever. Notably, we saw a decrease in the incidence of fever in the treatment group [19]. Despite the apparent reduction in the incidence of fever among the group that received prophylactic antibiotics, we observed that patients with a life expectancy of fewer than 90 days were excluded from the study. This could account for the amplification of the effect of the intervention on the treatment group. The largest clinical trials enquiring about our area of concentration were the preventive antibiotics in stroke study (PASS) trial and Stroke-INF trial. The latter, however, focused more on the incidence of post-stroke pneumonia in patients with dysphagia secondary to stroke. This particular trial was cluster-randomized, making selection bias a major limiting factor. The primary outcome evaluated by the PASS trial was the functional outcome of the patients that received prophylactic antibiotics after three months post-stroke. The study, although an open-label randomized control trial, employed the use of blinded evaluators. The assessment was done using the modified Rankin scale (mRS). There was no difference between both groups in respect to the primary outcome after three months. Still, there was a decrease in the infection rate (18% vs. 10%) as a secondary outcome. Interestingly, the incidence of post-stroke pneumonia remained the same. There was, however, a reduction in the incidence of urinary tract infections. The criteria for diagnosing urinary tract infections weren't clear in both studies, including how they were able to distinguish a urinary tract infection from asymptomatic colonization or stratification according to catheter practice since it was a multi-center trial. We can examine the effect prophylactic antibiotics had on infections in both trials in Table 4 below.

**Table 4:** The effect of prophylactic antibiotics after an acute stroke on the overall infection rate and incidence of urinary tract infections in the PASS and Stroke-INF trials comparatively PASS = preventive antibiotic in stroke study

Trial	Overall infection rate	Rate of urinary tract infections
PASS [13]	Reduced	Reduced
Stroke-INF [14]	Same	Reduced

Among patients receiving treatment intervention, the patients who received Intravenous thrombolysis did relatively better. The Stroke-INF trial was a large trial involving 48 stroke units. Similar in design to the PASS trial (open-label randomized control) primary outcome was the incidence of pneumonia in patients with dysphagia (dysphagia onset <48 hours after stroke onset). One could argue that patients with dysphagia secondary to stroke are severe and do not offer a representative view on the efficacy of preventive antibiotics for the entire population suffering from an acute stroke.

In summary, the risk of developing an early infection (< 7 days) was significantly reduced among the antibiotic group in all five studies [13,14,17-19]. The incidence of urinary tract infections in all five studies was also reduced, but only the Stroke-INF trial did not show any reduction in the onset of early pneumonia [13,14,17-19].

Functional outcome

Post-stroke infections have been implicated with mortality and poor functional outcome [20,21]. This was the primary outcome that was evaluated in the PASS trial. Both groups were to be accessed after 180 days via the mRS. The selection bias remains due to the design of the study. Even though the evaluators were blinded, a performance bias still can't be ruled out as patients were aware of what group they belonged to. Unsurprisingly there was no difference in functional outcomes in both groups [13]. Similarly, functional outcome was also enquired in the Stroke-INF trial, albeit as a secondary outcome with similar results [14]. Another additional issue is the use of the mRS as an assessment tool due to its subjectivity and reproducibility as limitations [22]. Still, both studies respectively portrayed similar distribution in each level of the mRS [13,14]. One may also ask if the 90 days used in the Stroke-INF are enough to judge the functional outcome in stroke patients even though the mRS was used in both studies. The preventive antibacterial therapy in acute ischemic stroke trial (PANTHERIS trial) by Harms et al. gives us a picture of this attribute after 180 days. However, this trial was not equipped enough to provide a definitive answer to the functional outcome. It concluded that stroke infections and a poor neurological outcome might not be related causally [18]. Post-stroke infections may however be a modality of inferring about the future neurological outcome in our patients. Schwarz et al. was the only study to see some degree of positivity in functional outcome, with a combined patient population of 60. It should be noted that this was a secondary outcome that was not blinded during measurement [19]. This study is limited most likely by observational bias from the researchers and its minute patient load.

Mortality

Post-stroke infections have been associated with unfavorable outcomes; although the trials carried out so far have not given any evidence towards considering its inclusion into our stroke guidelines, a mortality benefit might be the missing piece to justify its inclusion. PASS trial and Stroke-INF had no difference in mortality rate between the treatment and standard of care groups [13,14]. One outstanding feature about both trials was the way they stratified each group according to their respective co-morbidities. However, in our opinion, the PASS trial was more specific to this regard as daily drugs like statins were taken into account. Nevertheless, superficially one may say that the Stroke-INF trials had more severe patients when compared with the PASS trial. The latter trial included patients with a National Institute of Health Stroke Scale of greater to or equal to one, and the patients predominantly in the study had a mild stroke. It appears that both studies could tentatively be representative of the extremes of the severity of the stroke population. Could this be why there was no mortality benefit picked up in either study? Other systematic reviews and meta-analyses have been done. They have concluded that prophylactic antibiotics may reduce the incidence of infections after a stroke but proffered no benefit in improving functionality and mortality [4,23-26].

Looking holistically, we lack empiric evidence on the effect of antibiotics on major outcomes that influence patient life (functional ability and mortality). We should look at post-stroke infections more as a measure of the severity and prognosis in stroke patients because of available data. It seems that the more severe the stroke, the more the chances of acquiring a post-stroke infection.

Limitations

The following factors may have limited this study to an extent. These include true quality of the primary studies involved, inconsistent time frames for initiation and measurements of interventions and outcomes, heterogeneity of inclusion criteria, different NIHSS benchmarks, inconsistent use of either only ischemic or both Ischemic and hemorrhagic stroke.

## Conclusions

After an extensive analysis of the above clinical trials and systematic reviews, we found that antibacterial agents play no role in the outcome of patients who have suffered a stroke. They may reduce the onset of early infections, particularly urinary infections but have no role in the prophylaxis against post-stroke pneumonia. Also, the possibility of complicating our fight against anti-microbial resistance cannot be neglected if we continue to consider empiric prophylactic antibiotics. This proves that more focus should be put into improving stroke unit care, more research in interrupting the various immunomodulatory mechanisms that occur after a stroke, and preventive medicine. We believe the exclusion of prophylactic antibiotics in our current guidelines is justified based on available evidence. However, more studies with better designs should be carried out to identify any particular cohort that may benefit from prophylactic antibiotics.

## References

[1] Westendorp WF, Nederkoorn PJ, Vermeij JD, Dijkgraaf MG, van de Beek D (2011). Post-stroke infection: a systematic review and meta-analysis. BMC Neurol.

[2] McCulloch L, Smith CJ, McColl BW (2017). Adrenergic-mediated loss of splenic marginal zone B cells contributes to infection susceptibility after stroke. Nat Commun.

[3] Vermeij FH, Scholte op Reimer WJ, de Man P (2009). Stroke-associated infection is an independent risk factor for poor outcome after acute ischemic stroke: data from the Netherlands Stroke Survey. Cerebrovasc Dis.

[4] Liu L, Xiong XY, Zhang Q, Fan XT, Yang QW (2016). The efficacy of prophylactic antibiotics on post-stroke infections: an updated systematic review and meta-analysis. Sci Rep.

[5] Teh WH, Smith CJ, Barlas RS (2018). Impact of stroke-associated pneumonia on mortality, length of hospitalization, and functional outcome. Acta Neurol Scand.

[6] Learoyd AE, Woodhouse L, Shaw L (2017). Infections up to 76 days after stroke increase disability and death. Transl Stroke Res.

[7] Liu DD, Chu SF, Chen C, Yang PF, Chen NH, He X (2018). Research progress in stroke-induced immunodepression syndrome (SIDS) and stroke-associated pneumonia (SAP). Neurochem Int.

[8] Net P, Karnycheff F, Vasse M, Bourdain F, Bonan B, Lapergue B (2018). Urinary tract infection after acute stroke: impact of indwelling urinary catheterization and assessment of catheter-use practices in French stroke centers. Rev Neurol (Paris).

[9] Smith CE, Schneider MA (2020). Assessing postvoid residual to identify risk for urinary complications post stroke. J Neurosci Nurs.

[10] Stanley D, Mason LJ, Mackin KE (2016). Translocation and dissemination of commensal bacteria in post-stroke infection. Nat Med.

[11] Maier IL, Becker JC, Leyhe JR, Schnieder M, Behme D, Psychogios MN, Liman J (2018). Influence of beta-blocker therapy on the risk of infections and death in patients at high risk for stroke induced immunodepression. PLoS One.

[12] Yuan M, Han B, Xia Y, Liu Y, Wang C, Zhang C (2019). Augmentation of peripheral lymphocyte-derived cholinergic activity in patients with acute ischemic stroke. BMC Neurol.

[13] Westendorp W, Vermeij J, Zock E (2015). The Preventive Antibiotics in Stroke Study (PASS): a pragmatic randomised open-label masked endpoint clinical trial. The. Lancet.

[14] Kalra L, Irshad S, Hodsoll J (2015). Prophylactic antibiotics after acute stroke for reducing pneumonia in patients with dysphagia (STROKE-INF): a prospective, cluster-randomized, open-label, masked endpoint, controlled clinical trial. Lancet.

[15] Moher D, Liberati A, Tetzlaff J, Altman DG, PRISMA Group (2009). Preferred reporting items for systematic reviews and meta-analyses: the PRISMA statement. PLoS Med.

[16] .Shea BJ, Reeves BC, Wells G (2017). AMSTAR 2: a critical appraisal tool for systematic reviews that include randomised or non-randomised studies of healthcare interventions, or both. BMJ.

[17] Chamorro A, Horcajada JP, Obach V (2005). The early systemic prophylaxis of infection after stroke study: a randomized clinical trial. Stroke.

[18] Harms H, Prass K, Meisel C (2008). Preventive antibacterial therapy in acute ischemic stroke: a randomized controlled trial. PLoS One.

[19] Schwarz S, Al-Shajlawi F, Sick C, Meairs S, Hennerici MG (2008). Effects of prophylactic antibiotic therapy with mezlocillin plus sulbactam on the incidence and height of fever after severe acute ischemic stroke: the Mannheim infection in stroke study (MISS). Stroke.

[20] Rohweder G, Ellekjær H, Salvesen Ø, Naalsund E, Indredavik B (2015). Functional outcome after common poststroke complications occurring in the first 90 days. Stroke.

[21] Popović N, Stefanović-Budimkić M, Mitrović N (2013). The frequency of poststroke infections and their impact on early stroke outcome. J Stroke Cerebrovasc Dis.

[22] Broderick JP, Adeoye O, Elm J (2017). Evolution of the modified Rankin scale and its use in future stroke trials. Stroke.

[23] Xi YG, Tian X, Chen WQ (2017). Antibiotic prophylaxis for infections in patients with acute stroke: a systematic review and meta-analysis of randomized controlled trials. Oncotarget.

[24] Han X, Huang J, Jia X, Peng L, Yan K, Zan X, Ma L (2018). Preventive antibiotics for poststroke infection in patients with acute stroke: a systematic review and meta-analysis. Neurologist.

[25] Zheng F, Spreckelsen NV, Zhang X (2017). Should preventive antibiotics be used in patients with acute stroke? A systematic review and meta-analysis of randomized controlled trials. PLoS One.

[26] Rashid MH, Kabir A, Waris MU, Salman U, Zain S (2020). Role of prophylactic antibiotics in critical care of stroke patients - a preventive approach to post-stroke infections?. Cureus.

